# Requirement of epithelial integrin-linked kinase for facilitation of *Citrobacter rodentium*-induced colitis

**DOI:** 10.1186/1471-230X-13-137

**Published:** 2013-09-11

**Authors:** Kiran Assi, Kirk Bergstrom, Bruce Vallance, David Owen, Baljinder Salh

**Affiliations:** 1 Division of Gastroenterology, Department of Medicine, The University of British Columbia, 5th Floor, 2775 Laurel Street, V5Z 1M9 Vancouver, BC, Canada; 2 Child and Family Research Institute, Vancouver, Canada; 3 Anatomical Pathology, Vancouver V5Z 1M9, BC, Canada

**Keywords:** ILK, Citrobacter, Colitis, Fibronectin

## Abstract

**Background:**

Integrin-linked kinase (ILK) is a serine-threonine kinase that transduces extracellular matrix-related cues into intracellular signals, with fundamental roles in cell motility, development and cancer. Recently ILK been shown to have an important role in bacterial epithelial cell attachment, through ILK-bacterial OspE binding. Here we report on the role of epithelial derived ILK in response to *Citrobacter rodentium* infection.

**Methods:**

*C. rodentium* was administered to both control and intestinal epithelial cell ILK knockout mice. Histological inflammatory scores were assessed, and cytokines measured by ELISA as well as RT-PCR, in mouse colons. Bacterial colonization was determined by plating homogenates onto MacConkey agar, and immunofluorescence microscopy performed using anti-LPS and anti-Tir antibodies.

**Results:**

ILK-ko mice exhibited reduced weight loss at 15 days post-infection (p < 0.01) and demonstrated reduced histological inflammatory scores (p < 0.01), reduced CCL2 and pro-inflammatory cytokines. This was not due to reduced colonization, but was associated with an altered pattern of *C. rodentium* bacterial migration. Attenuated fibronectin expression was found in the ILK-ko mice. *C. rodentium* exposure was shown to increase ILK expression in cell lines, and in murine epithelium *in vivo*. In ILK-ko mice reduced activation of ser473Akt and reduced crypt proliferation, together with reduced cyclin D1 expression were observed.

**Conclusions:**

ILK influences the host response to *C. rodentium* -induced infection, independently of reduced colonization in the ILK knockout mice. The reduced inflammation and dramatically attenuated hyperplastic cryptal response to infection in this group, are at least in part the result of, the reduction in CCL2 and cyclin D1 expression respectively.

## Background

Gastrointestinal infection is an important cause of mortality in the developing world and morbidity in the developed world [1,2]. Although a variety of bacteria and viruses are known to cause gastroenteritis, the underlying mechanisms involved remain unknown. Several barriers are known to mitigate against intestinal infection and these include physical defenses such as the surface mucus layer, cell-cell junctions, rapid epithelial cell turnover, the presence of commensal bacteria, as well as the innate immune system responsible for the production of immunoglobulin A, defensins and resident immune cells [3,4]. Infection occurs when organisms are successfully able to breach these barriers. Some of the most important organisms causing bacterial infection world-wide are Enteropathogenic and enterohaemorrhagic Escherichia coli (EPEC and EHEC respectively). An organism that is useful to study mechanistic aspects of this process is *C. rodentium*, colonization by which results in epithelial injury through the development of development of F-actin-rich pedestals, otherwise known as attaching and effacing lesions in mice [5,6]. This process is known to rely on a type III secretion system used to inject bacterial effectors into host epithelium. Due to similarities with human idiopathic inflammatory bowel disease, such as a predominant Th_1_ response, attended by the elaboration of cytokines such as interferon gamma, the *C. rodentium* model has also been used to investigate mechanisms involved in that group of disorders.

ILK was first discovered as a beta 1 (β1) integrin binding protein via a yeast 2-hybrid assay. Since then it has been shown to play an important role in focal adhesion formation, which it achieves by complexing with Pinch and the Parvin proteins [7]. A significant body of work has shown that ILK plays a role in tumor biology. Other work indicates a role in cardiac development, blastocyst implantation, skin, connective tissue, hepatic and gut development [8].

ILK has been shown to be involved in the uptake of *Streptococcus pyogenes* and other bacteria into epithelial cells [9]. More recent work has shown that host intestinal ILK may be subverted by *Shigella flexnerii* in order to stabilize focal adhesions [10]. This facilitates blocking of cell detachment and hence the bacteria are able to gain a foothold for infection to proceed. An effector protein OspE, which is conserved in enteropathogenic *E. coli*, *Salmonella* and *C. rodentium*, was shown to bind to ILK and co-localize with it at focal adhesions.

It is presently unclear exactly how ILK within the gastrointestinal epithelium may modulate the binding of, and response to infection with organisms such as *C. rodentium*. In our previous work we have shown that conditional knockout of ILK in epithelial cells blunts the response to inflammation-induced cancer development in the colon [11]. We have also demonstrated that epithelial ILK deficiency leads to attenuation of DSS-induced colitis, an effect that was associated with a reduction in fibronectin expression, as well as an alteration in the ratios of lymphocyte populations [12]. In this work we have investigated how ILK deficiency affects the host response to *C. rodentium* infection.

## Methods

Anti-Tir and anti-LPS antibodies were obtained from Dr B Vallance; anti-fibronectin antibody from Abcam (Cambridge, MA); ILK, Akt, Gadph, actin, cyclin D and Ki-67 antibodies from Santa Cruz (Santa Cruz, CA), ser473 Akt from Cell signaling, ILK si-RNA from Qiagen, Akt and ILK antibodies from Santa Cruz; anti-CD3. ELISA kits for TNFα, IFNγ, IL-10, CCL2 were obtained from BD Biosciences (Mississauga, ON). Horse-radish peroxidase conjugated secondary antibodies were obtained through Calbiochem (San Diego, CA). EGTA, EDTA, MOPS, PMSF, sodium orthovanadate, leupeptin, aprotinin, benzamidine, dithiothreitol and β-glycerolphosphate, were purchased from Sigma.

### ILK-ko mice, disease activities

We have previously described our ILK knockout mice [11]. Briefly, mice on an FVB (Friend virus B-type) background were kept in conventional housing in the animal care facility at Jack Bell Research Centre. They were fed chow ad libitum and had liberal access to drinking water. All experiments were approved by the UBC Animal Ethics Committee (A05-1580). Inactivation of ILK in colonic epithelial cells was achieved by crossing the Fabp –Cre mice with ILK^flox/flox^ animals. The resulting offspring were then backcrossed with the homozygote floxed mice to generate the genotype: ILK^flox/flox^,Cre. Genotyping for Cre and ILK were carried out as previously described. Briefly, tail DNA was obtained and the following primers used to detect Cre expression: 5′–CCTGGAAAATGCTTCTGTCCG–3′ and 5′- CAGGGTGTTATAACAATCCC-3′. ILK genotype was monitored using: 5′- CCAGGTGGCAGAGGTAAGTA-3′ and 5′-CAAGAAATAAGGTGAGCTTCAGAA-3′.

For infection experiments mice were used out at approximately 6 to 8 weeks of age. C rodentium (strain DBS 100) was given by gavage as an inoculum of 2.5 × 10^8^ bacteria per 100 ul of Luria broth and the mice terminated either on day 6 or 15.

Their colons were examined for macroscopic and, using hematoxylin and eosin, for microscopic disease activity as previously described with some modifications [13]. After removal, the colons were fixed in 10% buffered formalin for immunohistochemistry, protein lysates were prepared for western analysis and ELISA. With reference to disease activity scoring: A. Macroscopic assessment of disease activity was scored from 0–4 as follows: 0, no signs of inflammation, normal pellet, and from 1–4 depending on the degree (slight, moderate, severe) of liquidity of stool, presence of hyperemia and thickening of the distal bowel, presence of blood, as well as the degree of weight loss. B. Microscopic scores were scored by a gastroenterological pathologist with experience in murine mucosal pathology (D Owen). This score grades the severity of the lesion from 0–4, based on the severity of inflammation, the extent of inflammation (depending on mucosal → transmural inflammation), ulceration, crypt damage, and percentage involvement observed across 5 different microscopic fields per mouse.

### SDS-polyacrylamide gel electrophoresis

Western immunoblotting was performed as previously described [12]; colonic tissue or cultured cells were homogenized in buffer containing 20 mM MOPS, 150 mM NaCl, 50 mM β-glycerophosphate, 5 mM EGTA, 50 mM NaF, 1 mM DTT, 1 mM sodium vanadate, 0.5% NP40 and 1 mM PMSF. After sonicating for 15 s (×2) and centrifuging at 14,000 RPM for 15 min, the protein concentration in the supernatant was determined by the Bradford assay (Bio-Rad, Mississauga, Ont). 25 ug of protein from each sample was resolved using 10% SDS-PAGE before transferring to nitrocellulose membranes (Bio-Rad). The blots were blocked in 5% skim milk in TBST (20 mM Tris–HCl pH 7.4, 250 mM NaCl, 0.05% Tween-20) for 1 h before probing for 2 h using the appropriate primary antibody. The blots were washed with TBST for 10 min three times, before being incubated with the appropriate secondary antibody for 1 h. Following 3 further washes in TBST, they were developed using the enhanced chemiluminescence detection system (ECL, Amersham, Montreal, Quebec).

### Immunohistochemistry

Paraffin-embedded colonic tissue samples were de-waxed in xylene twice for 5 min, rehydrated in a series of ethanol (100% - 70%) for 3 min each followed by rehydration in PBS for 30 min. After rehydration the endogenous peroxidase was blocked with 0.3% hydrogen peroxide followed by antigen retrieval by microwaving sections in citrate buffer pH 6.0 (10 mM Na citrate). Following antigen retrieval, the sections were washed three times with PBS, blocked in 1% BSA for 1 h, and then stained using the Vectastain ABC kit (Vector laboratories, Burlingame, CA) mentioned below according to manufacturer’s recommendations but with the following modifications. Sections were incubated with the following primary antibodies at 4°C overnight: cyclin D1 (Santa Cruz, CA), fibronectin (1:200, Abcam, Cambridge, MA), Ki-67. Following incubation, the sections were rinsed three consecutive times with PBS and then incubated with the appropriate biotinylated secondary antibody for 1 h followed by incubation with peroxidase-labelled streptavidin. Nova –red and DAB were used as the chromagens, and the sections were counterstained with haematoxylin. Three blinded observers independently examined all stained sections [12].

For detection of Tir and LPS by immunofluorescence, the slides were processed as for IHC and the following antibodies were used: Tir and LPS (Vallance). Sections were stained with Vectastain ABC elite kit and biotinylated ant-rabbit for DAPI, or eFluor650 Nanocrystal conjugation kit, cat no. 88-7072-98 antibody, and Avidin D-FITC (or Avidin-Texas Red) used for immunofluorescence (Vector laboratories, CA, USA). A Zeiss LSM-780 microscope was used to capture images. Each section had its own control using the secondary antibody only. Pre-immune serum was initially used to ensure specificity of the signal with each of the antibodies.

### Cell culture

HCT 116 cells were a kind gift of Bert Vogelstein (Johns Hopkins, Baltimore, Maryland) and were cultured in McCoys 5A Medium (Gibco, Burlington, Ontario) containing 10% heat inactivated fetal bovine serum (FBS) (Hyclone, Logan, Utah). Protein lysates were obtained using homogenization buffer as described above.

CMT93 cells are derived from a murine rectal cancer and were obtained from B Vallance (Vancouver, BC). They were cultured in DMEM containing 10% FBS and 2 mM glutamine; experiments were performed when cells were approximately 90% confluent.

### Semiquantitative RT –PCR

1 ug of RNA, obtained using Trizol from HCT 116 cells (or murine colon), was reverse transcribed using random hexamers (Perkin-Elmer Applied Biosystems, Branchburg, NJ) and 20 units of Moloney murine leukemia virus reverse transcriptase M-MLV (Invitrogene) in 20 μl of total volume at 25°C for 10 min and at 37°C for 60 min. The resulting first-strand complementary DNA (cDNA) was used as template for the semi- quantitative-PCR. Amplification of the following cDNAs was performed using the primers listed: CCL2 (F):ATGCAGGTCCCTGTCATGCTTCTG; (R):CTAGTTCACTGTCACACTGGTCACTCC; b-actin(F):AGAGGGAAATCGTGCGTGAC; (R):CAATAGTGATGACCTGGCGGT. Relative quantification of gene expression was performed using densitometry and beta-actin as a control.

### Si-RNA-mediated knockdown of ILK

This was performed as described previously using a 21-mer to transfect HCT116 cells, grown to 60% confluency, using Silentfect (Biorad). Two separate ILK si-RNA and control (scrambled) sequences were purchased from Qiagen Inc (Mississauga, ON), and from Santa Cruz Biotechnology Corporation Inc (Santa Cruz, Ca). Gene knockdown was confirmed using western blotting and Q-PCR.

### Bacterial counts

After homogenization of either cecal or colonic tissue, or stool pellets, they were serially diluted. They were then plated onto MacConkey agar plates and bacterial colonies were enumerated after 1 day [14].

### Statistical analysis

All macroscopic and histological disease scores, as well as cytokine levels were expressed as mean + SD, with p < 0.05 being considered significant using the Student’s t-test (unpaired, two-tailed). Where indicated ANOVA was performed with Tukey post-hoc testing.

## Results

### *C. rodentium* induces ILK and activates Akt in epithelial cells

**Figure 1:**
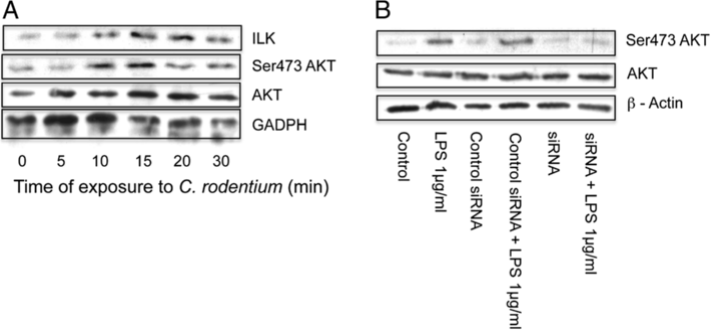
**ILK is induced in response to *C. rodentium *exposure of CMT 93 cells, together with activation of Akt, and ILK regulates Akt activation in response to LPS. A**. Colonic CMT 93 cells were exposed to *C. rodentium* for the times indicated. Cellular protein was obtained by lysing cells in homogenization buffer, and western blotting performed for the molecules indicated. **B**. After knocking down ILK using si-RNA (or scrambled control oligo) HCT116 cells were stimulated with LPS (1 ug/ml) for 30 min and western immunoblotting performed. GAPDH and β-actin were used as the loading controls.

Our first objective was to investigate if bacterial exposure of epithelial cells led to any change in the levels of expression of ILK. CMT 93 cells were exposed to *C. rodentium* and cells were harvested at the time points shown (Figure 1A). As the data indicates this led to an increase in the expression of ILK without affecting the levels of Akt, an important kinase involved in cellular survival. However there is a clear increase in the intensity of the ser473 Akt signal, the site known to be critically involved in its activation. In order to address whether ILK was responsible for the activation of Akt we used HCT 116 cells. We have found that these cells respond reliably to LPS and have been able to knock down ILK using si-RNA in this system. The data (Figure 1B) shows that knockdown of ILK attenuates the response of these cells to LPS induced ser473 Akt activation. Collectively these findings indicate that epithelial cells have the capacity to activate Akt via ILK.

### *C. rodentium* –induced colitis is attenuated in ILK-ko mice, and ILK is induced in response to infection

**Figure 2:**
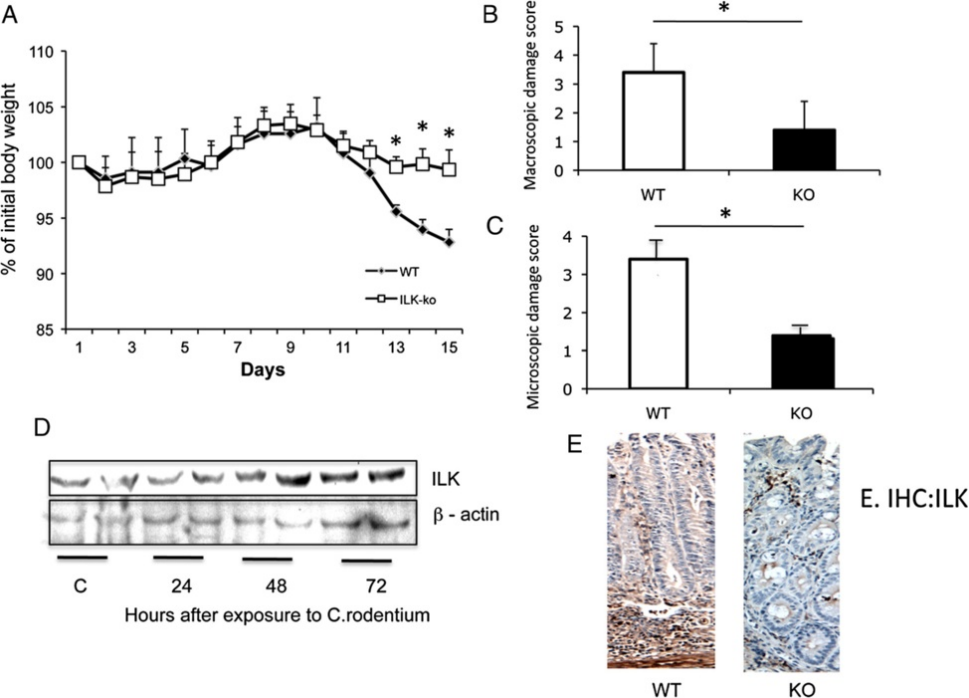
**ILK-ko mice are protected from *C. rodentium *induced wasting disease and inflammation, and exhibit reduced expression of the chemokine CCL2. A**. Colitis was induced in 6 animals per group by administering *C. rodentium* by gavage. Animal weights were recorded daily. There was an observed significant difference in their weights from days 13–15 (p < 0.05). **B**. Assessment of macroscopic damage in ILK-ko mice (day 15) indicated a significant reduction in comparison to control mice (**p < 0.01). **C**. Microscopic damage scores (day 15) were determined using criteria outlined in Materials and methods, based on severity and extent of inflammation. The data indicate there was reduced damage in the ILK-ko mice. **D**. ILK expression was assessed in response to Citrobacter at the time points indicated using western immunoblotting. The representative data indicated an increase in ILK expression after 48–72 hours (b-actin is shown as a loading control). **E**. ILK immunohistochemistry is shown 15 days post-infection in a wild-type and ILK-ko sample. There is a reduction in the ILK signal intensity in the epithelial cells as compared with the comparable intensity in the infiltrating immune cells.

The *C. rodentium* induced murine colitis model is a very well characterized system to investigate host-microbial interactions, as well as the ensuing inflammatory response. A mild colitis usually results after 10 to 15 days post-infection, which is usually accompanied by a mild degree of weight loss [15], however a fatal form of the disease, characterized by severe inflammation has been described in the FVB strain [16]. We investigated this in our ILK-ko mice in comparison with their littermate control mice. The data (Figure 2A) obtained from six mice per group, indicates that the weight-loss response is attenuated in the ILK-ko mice. In Figure 2B there is a clear reduction of macroscopic inflammation in the knockout mice. Similar changes were seen at the level of histological inflammation (Figure 2C). In order to verify that there was an induction of ILK protein in response to *C. rodentium in vivo* we performed a time course experiment. Using mucosal scrapes from mice terminated at days 1–3, and western immunoblotting, we observe an increase in ILK at between 2–3 days (Figure 2D). A comparison of the levels of ILK present in the 2 sets of mice used in these studies, shows a difference at the end of 15 days (Figure 2E). Whilst both panels show positive staining within the immune cells coursing between the crypts, there is a clear reduction of the ILK signal from the epithelial cells, in the representative ILK-ko example shown.

### *C. rodentium* induces CCL2 and macrophage infiltration; both of which are blunted in ILK-ko mice

**Figure 3:**
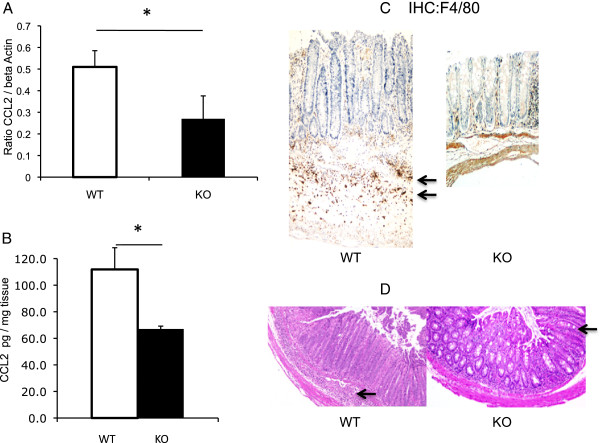
**ILK-ko mice exhibit reduced expression of the chemokine CCL2, and reduced monocyte-macrophage influx. A**. CCL2 induction (RT-PCR, message compared to that of β-actin) was significantly (**p < 0.01) reduced in the ILK-ko group. **B**. Distal colonic lysates (pooled) were used to determine CCL2 levels by ELISA. The data are for 6 mice per group and show a reduction in levels of CCL2 in the ILK-ko mice (*p < 0.01). **C**. IHC for F4/80 which is a marker for cells of the monocyte/macrophage lineage shows reduced numbers in the ILK-ko mice. **D**. Histology of a representative section from each of the WT and ILK-ko mice shows less inflammation and hyperplasia in the ILK-ko example shown. Notably, there are elongated crypts seen in the ILK-ko mice as can be seen to the right side of the ILK-ko panel.

We next characterized the levels of expression of CCL2 in these groups. We have shown that this chemokine is reduced in another model of colitis induced by dextran sodium sulfate. As we have previously shown that ILK may impact on the level of expression of CCL2 expression by RT-PCR in an epithelial system, this was investigated in this model also. Similar to our findings in the DSS-induced colitis, we showed a reduction of CCL2 in the ILK-deficient mice, both at the level of message and protein (Figure 3A/B). To determine whether or not this was associated with any change in infiltration of cells of the monocytic series, we performed immunohistochemistry with the F4/80 antibody, which recognizes monocytes and macrophages. The data clearly shows an impressive number of cells in the submucosa of the wild-type mice, which are missing in the ILK-ko example shown. Typical histological sections (Figure 3D) show the crypt hyperplastic response together with inflammation in the wild-type mouse, which is blunted in the ILK-ko section shown.

### ILK-ko mice have reduced levels of pro-inflammatory cytokines

**Figure 4:**
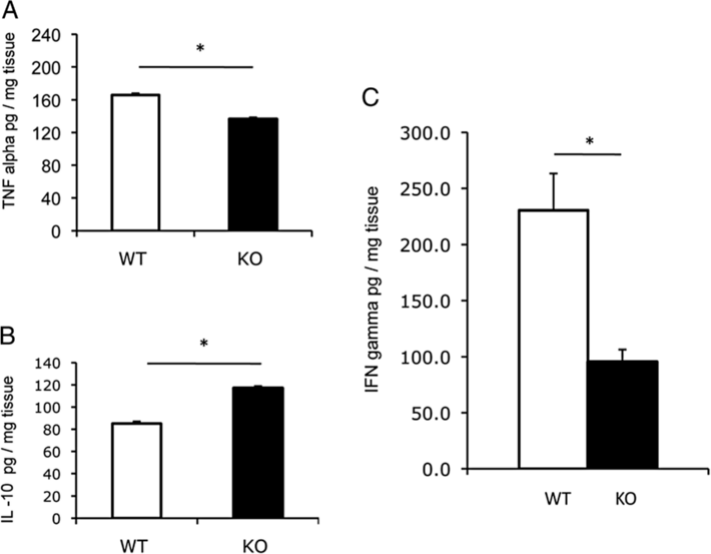
**ILK-ko mice express reduced pro-inflammatory cytokines. A**-**C**. Distal colonic lysates (pooled) were used to determine TNF alpha, IL-10 and IFN gamma levels by ELISA. The data are for 6 mice per group and show a reduction in levels of TNF alpha and IFN gamma, as well as an increase in IL-10 in the ILK-ko mice (*p < 0.05).

Measurement of key cytokines, representative of pro- and anti-inflammatory effectors, tumor necrosis factor alpha (TNFα), interferon gamma (IFNγ) and interleukin 10 (IL-10), revealed that there were significant reductions in TNFα and IFNγ in the knockout mice, which was associated with an increase in the level of IL-10 (Figure 4A-C). Overall this data indicates that ILK deficiency specifically in the epithelial cell component, directly or indirectly, is associated with a reduced inflammatory cytokine response due to *C. rodentium*.

### ILK-ko mice have diminished Akt activation in response to *C. rodentium*

**Figure 5:**
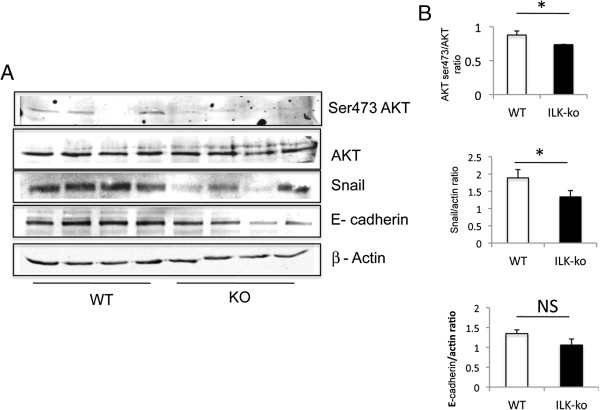
**ILK-ko mice have reduced Akt activation, and reduced expression of Snail, but not E-cadherin after *C. rodentium *induced colitis. A**. Lysates prepared from distal colonic homogenates were resolved using Western analysis and the resulting membranes probed with the antibodies indicated (4 mice from ILK-ko and littermate controls). **B**. Densitomtry was performed and the resulting data is depicted in the barcharts (Akt, Snail and E-cadherin).

Next we wanted to assess the impact of inflammation on the expression of key ILK regulated proteins in the intestine. In accordance with the data in the cell lines, we observed an attenuation of the activation of Akt in the ILK-ko mice, as well as a reduction in the level of expression of the transcription factor Snail, but not E-cadherin, using western blotting (Figure 5A/B). Previous work has shown that expression of Snail and E-cadherin, can be modulated by ILK in epithelial cells [17]; ILK impacting on Snail expression thereby de-repressing E-cadherin expression. Furthermore we have shown a correlation between Snail and ILK-ko in a colitis - associated cancer model, where the ILK-ko mice had reduced expression of Snail in the neoplasms [11]. These findings indicate that genetic deletion of ILK in epithelial cells results in attenuation of the Akt activation response and Snail expression, although regulation of E-cadherin may be more complex.

### *C. rodentium* binding to apical epithelium is unimpaired in ILK-ko mice

**Figure 6:**
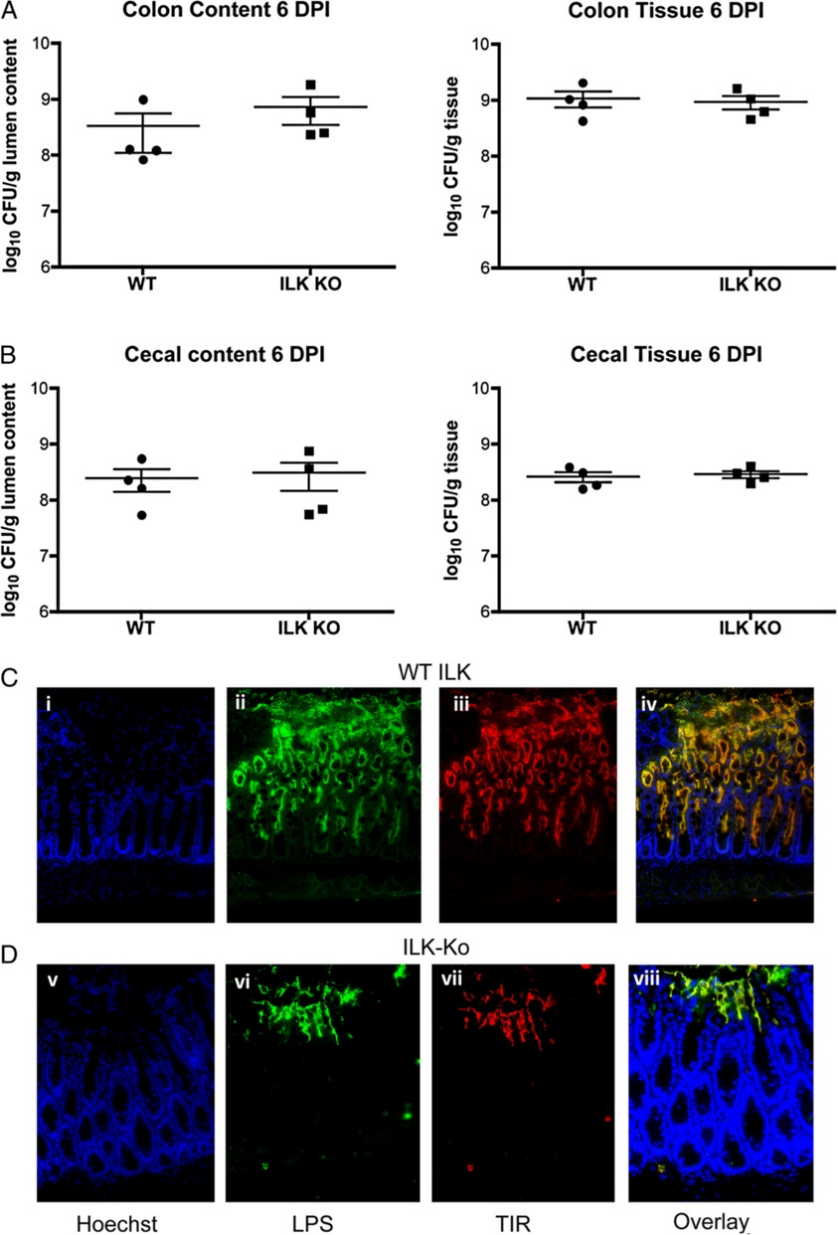
**Effects observed in ILK-ko mice are not due to impaired binding of *C rodentium*.** ILK-ko and control mice were gavaged with *C. rodentium* and their cecal and colonic tissues assessed for levels of *C. rodentium* by plating homogenates and counting the number of colonies, in both the colon **(A)** and cecum **(B)**. The tissues were also processed for immuno-fluorescence and probed with antibodies to LPS (green) and Tir (red), and overlayed (yellow). As can be seen bacteria are capable of binding in the ILK-ko mice but may be impaired in their ability to migrate downwards in between the crypts **(C**/**D)**.

Based on these findings we hypothesized that perhaps the blunted inflammatory and response to *C. rodentium* was due to impaired epithelial binding in the knockout mice. By employing a bacterial plating assay we measured the levels of bacteria in the cecum and colon, in both the luminal contents and in the mucosal lining. As the data in Figure 6 (A/B) indicate there appears to be no difference in the levels of bacteria in the two sets of mice, regardless of whether the colonic (or cecal) contents or tissue were examined. This clearly indicates that the responses observed must be occurring at or distal to the epithelial cell system in the mice, and importantly, that the responses were not simply due to a failure of the *C. rodentium* to bind.

### *C. rodentium* cryptal migration is impaired in ILK-ko mice

**Figure 7:**
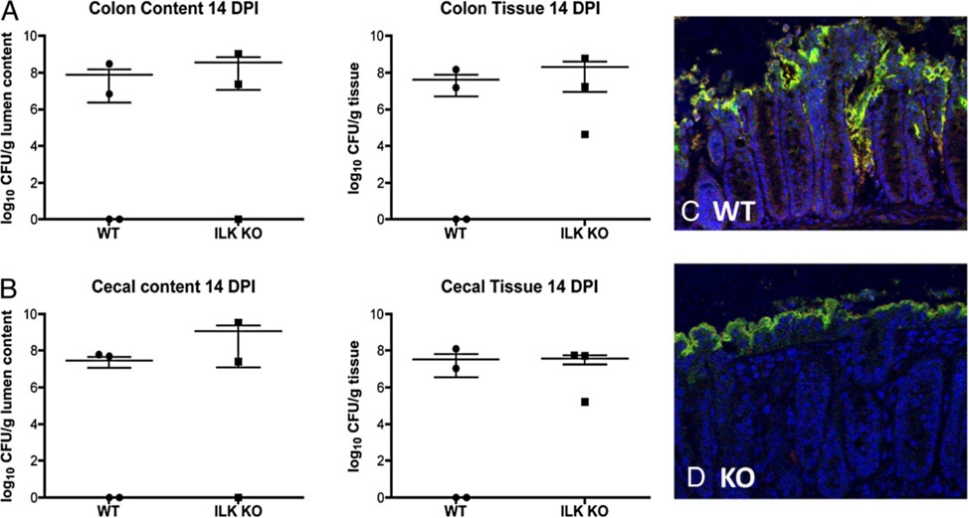
**Effects observed in ILK-ko mice are preserved at day 14 post-infection.** ILK-ko and control mice were gavaged with *C. rodentium* and their cecal and colonic tissues assessed (day 14) for levels of *C. rodentium* by plating homogenates and counting the number of colonies as for Figure 6**(A**/**B)**. The tissues were also processed for immuno-fluorescence in the same manner **(C**/**D)**. It can be seen that although there are still equivalent amounts of bacterial bainding, bacteria are still unable to migrate downwards in between the crypts, in the ILK-ko mice at this second time-point.

In order to verify that bacterial binding occurred in a similar distribution in the two sets of mice we performed immunofluorescence using previously described antibodies, one against *C. rodentium* LPS (green) and the other against TIR (red); the latter being a widely recognized method for demonstrating *C. rodentium* infection. The upper panels (Figure 6C) depict representative staining in the wild-type mice. As can be seen bacterial binding occurs at the apical surface and appears to migrate downwards along the lateral aspects of the crypts. In the lower panels for the ILK-ko mice (Figure 6D), it is evident that bacteria are able to bind to the apical region, however, there does not appear to be a comparable signal migrating downwards. This may be a factor in the reduced inflammatory response observed. In order to determine whether the response was being delayed in the ILK-ko mice we repeated the experiment at day 14. The data again show no significant differences in bacterial binding, or any increased migration at this later time-point (Figure 7A-C). This intriguing observation at 2 different time-points, indicates that an ILK-dependent mechanism, following bacterial binding, facilitates the intercryptal migration of bacteria.

### *C. rodentium* –associated hyperplasia is reduced in ILK-ko mice

**Figure 8:**
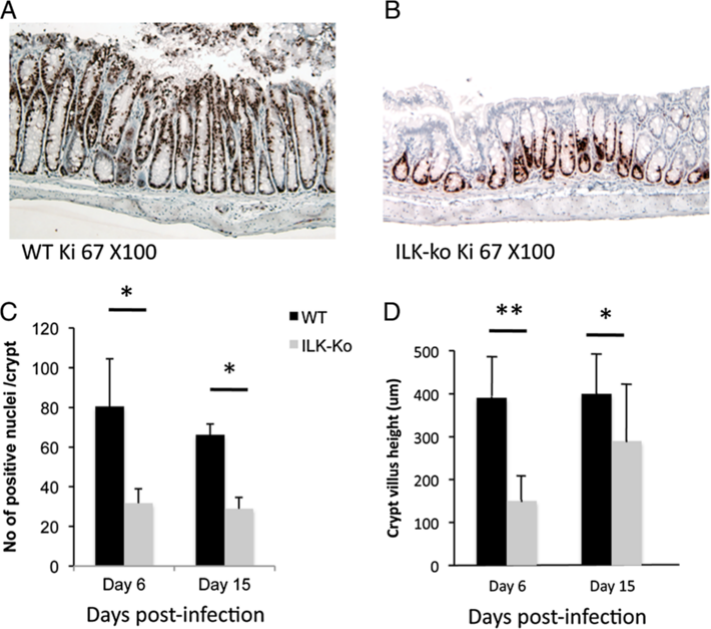
**Reduced proliferation in ILK-ko mice in response to *C. rodentium. *infection. A**, **B**. Sections were processed for immunohistochemistry using an antibody to Ki-67 at the time-points indicated. The photomicrographs are representative of data from 5 mice. **C**. The number of Ki-67 positive cells per crypt were counted in 5 HPFs at days 6 and 15. **D**. Crypt heights were measured using a micrometer in 5 different fields, in each of 5 mice per group.

As one of the key mechanisms involved in prevention of bacterial adherence by epithelia is related to increased epithelial cell turnover, we explored cellular proliferation using Ki-67 immunohistochemistry, in the ILK-ko and littermate control wild-type mice. As the representative pictures show there is clearly more enhanced proliferation in the wild-type versus the knockout mice (Figure 8A/B). The data in the barchart (Figure 8C) show the positive staining in the knockout mice is less than half of that seen in the wild-type mice. The crypt heights were measured and there was a clear reduction at both the 6 and 15 day time-points, in the ILK-ko mice. Interestingly, we noted an increase in the crypt height between the 2 time-points in the ILK-ko mice indicating that perhaps a delayed response to the bacteria was occurring. However as the data in Figure 8 indicate, there was no difference in the binding/distribution patterns at this later timepoint.

**Figure 9:**
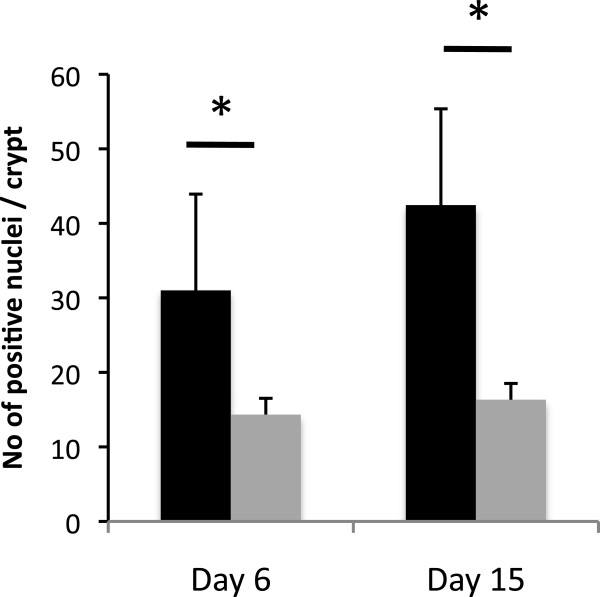
**Reduced cyclin D1 expression in ILK-ko mice in response to *C rodentium *infection.** Sections were processed for immunohistochemistry using an antibody to cyclin D1. The number of positively staining nuclei in 5 crypts per mouse in each of 5 mice were counted at days 6 and 15 (black: WT, grey:ILK-ko).

In order to determine the specific role of cyclin D1, a target of ILK, in this response we utilized immunohistochemistry. The data indicates (Figure 9) that the level of cyclin D is reduced in the ILK-ko mice, a finding in keeping with changes observed and previously reported by us in the ILK-ko mice in response to induction of cancer-associated colitis.

### Reduced *C rodentium* –induced fibronectin expression in ILK-ko mice

**Figure 10:**
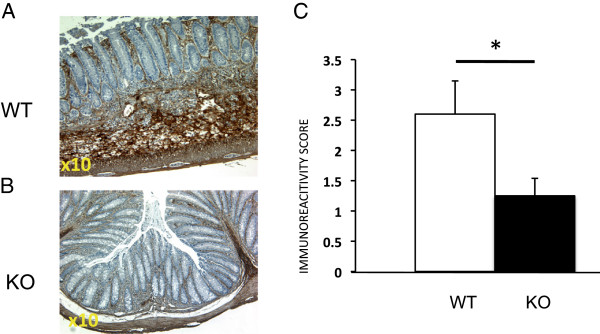
**Reduced fibronectin expression in response to C rodentium infection in ILK-ko mice.** After performing immunohistochemistry as described in Methods, fibronectin scoring was performed by examination of 5 HPFs in 4 mice from each group, using the following criteria: 0- no signal; 1- less than 10% of field, low intensity; 2-between 10-50% of field, moderate intensity; 3- over 50% of field, highest intensity. In the example shown, the ILK-ko has been scored as 1, and for the WT sample, 2 **(A**/**B)**. The overall data is shown in **C**.

The extracellular matrix may be an important determinant of bacterial ability to infect epithelial systems. In this regard a number of different bacteria utilize fibronectin to aid binding and/or invasion, whilst some are capable of expressing fibronectin-binding proteins [18]. As others, besides ourselves, have shown that ILK is involved in epithelial expression of fibronectin [19], we explored the possibility that this was the case in this system also. We have shown using immunohistochemistry that in the DSS-induced colitis model there is significantly less fibronectin expression in the ILK-ko mice. When we assessed this in the *C. rodentium* -induced colitis model we observe a similar finding, specifically that fibronectin expression is downregulated in the ILK-ko mice (Figure 10). This may be another mechanism to explain the reduced migration of the bacteria downwards between the crypts.

## Discussion

In this report we have added to our understanding of the role of ILK in intestinal pathophysiology, specifically in the setting of bacterial infection. Similar to the findings reported for DSS-induced colitis we show that there is a reduced inflammatory response, associated with a reduction in CCL2 expression, an important immune cell chemoattractant. Furthermore, our findings indicate that the pattern but not the magnitude of epithelial *C. rodentium* binding is altered in the ILK-ko mice, with preserved apical binding but reduced lateral epithelial cell binding/migration, in between adjacent crypts. Although the reduced expression of fibronectin may account for this finding, we cannot exclude alterations in other extracellular matrix components, or alterations in the levels of other cell surface integrins as being involved in this response. An additional role for ILK is indicated by the reduced crypt hyperplasia observed associated with decreased cyclin D1 on immunohistochemistry, in ILK-ko mice.

Bacteria utilize multiple mechanisms to gain a foothold facilitating their colonization, and several of these involve components of the extracellular matrix and integrin network [20]. The production of bacterial fibronectin-binding proteins (FnBPs) enables pathogens to bind host cell integrins through a fibronectin bridge. Organisms such as *Yersinia pseudotuberculosis* and *Shigella flexneri* undergo ingestion via integrin receptors. *Streptococcus pyogenes* uses M1 or PrtF1 surface proteins bound to fibronectin, to facilitate invasion via the α5β1 integrin receptor. As ILK interacts with the cytoplasmic domain of β1 integrin, this places ILK in a unique position to mediate downstream signaling. In the case of *C. rodentium* we can demonstrate equivalent levels of apical epithelial binding but downwards basolateral migration of the bacterium is impaired. This leads us to suspect that other receptors (possibly ILK-dependent integrins) are required for this. Future work will attempt to examine which integrins are specifically regulated by ILK in the intestinal epithelium.

OspE is a virulence factor common to several pathogens, including *C. rodentium*, EPEC, EHEC and *Salmonella* species, and is injected into host cells by the type III secretion system [21]. This has recently been shown to bind to ILK, resulting in an increase in the number of focal adhesions; OspE and ILK were shown to co-localize at focal adhesions through vinculin staining. It also caused an increase in the cell surface levels of β1 integrin, whilst at the same time reducing phosphorylation of focal adhesion kinase (FAK) and paxillin [10]. Together, this results in stabilization of focal adhesions and thereby facilitates bacterial cell adherence, through an attenuation of cell shedding. Our findings do not indicate a specific defect in bacterial adherence in ILK-ko mice, indicating that for *C. rodentium* this may not be a critical event. However there were clear differences in bacterial migration, indicating other (bacterial-mediated) ILK-dependent events that may be important. We cannot exclude the possibility that the effects we observe are the sum of two opposing events, the first due to a primary ILK-dependent reduction in cellular turnover thereby facilitating bacterial binding, and the second due to a reduction in ILK and OspE mediated reduction in bacterial binding.

Recent work has characterized the role of another important matrix protein, osteopontin (OPN) in the development of murine intestinal inflammation [22]. This has been previously shown to be upregulated in inflammatory bowel disease, but the exact significance of this observation remains unknown. In that work, OPN was induced in response to infection with *C. rodentium*, and it was noted that mice lacking OPN were colonized to a significantly reduced degree as compared with littermate controls. Consequently reduced pedestal formation and epithelial proliferation were observed and the former was reversed by the administration of human OPN. This indicates that bacteria have varying degrees of dependence on extracellular matrix components in facilitating their colonization, since apically at least we observed equivalent levels of binding (Figures 6 and 7), despite reduced fibronectin levels in ILK-ko mice.

Interestingly, infection with *C. rodentium* has not been associated with profound changes in apoptosis. This may be related to activation of the phosphatidylinositol 3-kinase (PI3K), a growth factor and TNFα -activated lipid kinase, which is associated with a cellular survival response. Using a pharmacological inhibitor Ly294002 it has been shown that PI3K is required for the host response for bacterial clearance, as well as the epithelial proliferative response [23]. This was reported to occur without any changes in inflammation. As previous work indicates that ILK is downstream of PI3K, some of our observations mirror these findings (reduced hyperplasia) whilst others may be dissociated from PI3K, most notably, the reduced inflammation in conjunction with the effect on the extracellular matrix.

Presently it is not known what specific molecules are involved in the sensing of epithelial damage and the resulting effectors of epithelial proliferation (and/or repair). The reduced epithelial proliferation consequent upon *C. rodentium* infection observed in our study may be due to 2 reasons. Firstly, because ILK is involved in the regulation of cyclin D1 this may be a direct effect at the level of the epithelial cell and independent of any bacterial-mediated mechanism [24]. As β-catenin is activated in response to C rodentium infection [25], and its casein kinase 1 (CK1) -mediated serine phosphorylation on residue 45 appears to coincide with hyperplasia [26], it is likely that cyclin D1 is activated directly in response to this. However in the FVB strain mice used in our work we were unable to demonstrate nuclear localization of β-catenin at either of the time-points investigated (days 6 or 15). The second reason may be an indirect one, and it is hypothesized to occur via the inability of the bacteria to migrate downwards in the ILK-ko in a similar manner to that observed in the wild-type mice.

Using Swiss Webster mice it has been shown that NF-κB is activated in response to infection with *C. rodentium*, and importantly that this is not correlated with the observed hyperplastic response. This was initially reported with the NF-κB inhibitor Velcade, which was demonstrated to inhibit NF-κB activation only [27]. More recently it has been shown that Mek, a component of the TLR4-Mek-MAPK pathway may mediate activation of NF-κB in vivo, demonstrated using the Mek1/2 inhibitor PD98059 [28].

Our study raises some important questions about the role of ILK in intestinal physiology and pathophysiology. We, and others have shown that ILK is upregulated at the protein level in intestinal and other tumors, indicating a role in tumorigenesis [29-31]. This is supported by a wealth of data regarding ILK’s role in various properties fundamental for cancer development such as proliferation, avoidance of apoptosis, angiogenesis and EMT. Our previous work using a colitis-associated cancer model showed a trend towards smaller tumors in ILK-ko mice that, was accompanied by a reduction in both cyclin D and Snail expression. This has been replicated in the model described in this report, which is also characterized by profound changes in cellular proliferation, indicating an important role for ILK in these two processes in the intestine. The reduction in Snail expression, which has been linked to EMT [32], in our ILK-ko mice is also of interest as the FVB strain of mice are known to undergo more fibrosis [15], and this is attenuated in the ILK-ko mice.

## Conclusions

Our findings indicate that *C rodentium* -induced colitis is impaired in mice lacking expression of ILK within the colonic epithelium. This appears to be dependent upon, or at least associated with, a reduction in epithelial proliferation as well as a reduction in inflammation. However, the observed effects do not appear to be related to impaired bacterial binding to the apical epithelium.

## Competing interests

The authors declare that they have no competing interests.

## Authors’ contributions

KA performed the cell culture and mouse studies, as well as the statistical analyses. KB carried out the bacterial counts. BS conceived and coordinated project and wrote the manuscript. DO carried out the histological assessments. BV helped with experimental design. All authors read and approved the final manuscript.

## Pre-publication history

The pre-publication history for this paper can be accessed here:

http://www.biomedcentral.com/1471-230X/13/137/prepub
